# Targeting Heparanase Attenuates Podocyte Injury Induced by Puromycin Aminonucleoside

**DOI:** 10.1002/jcp.70053

**Published:** 2025-06-10

**Authors:** Xing‐Yun Huang, Yu‐Hsien Lu, Hsiao‐Hui Lee

**Affiliations:** ^1^ Department of Life Sciences and Institute of Genome Sciences National Yang Ming Chiao Tung University Taipei Taiwan; ^2^ Center for Intelligent Drug Systems and Smart Bio‐devices (IDS2B) National Yang Ming Chiao Tung University Taipei Taiwan

**Keywords:** cytoskeleton, focal adhesion, heparanase, intercellular junction, podocyte injury, puromycin aminonucleoside

## Abstract

Podocytes are highly specialized glomerular visceral epithelial cells critical for maintaining the structure and function of the glomerular filtration barrier. These cells adhere to the glomerular basement membrane (GBM) and envelop the outer surfaces of the glomerular capillaries to prevent protein leakage during blood ultrafiltration. The GBM is a dense network of extracellular matrix composed of type IV collagen, laminin, nidogen, and heparan sulfate proteoglycans. In this study, we investigated the protective effect of a heparanase inhibitor on puromycin aminonucleoside (PAN)‐induced podocyte injury. Our results demonstrate that PAN treatment significantly disrupted the cytoskeletal architecture of cultured podocytes, reducing the formation of focal adhesions and stress fibers. Interdigitating intercellular junctions were replaced by dot‐like structures with accumulated filamentous actin. Co‐treatment with the heparanase inhibitor PI‐88 effectively prevented these PAN‐induced cytoskeletal abnormalities. Furthermore, a BSA filtration assay revealed that PI‐88 attenuated PAN‐induced increases in podocyte monolayer permeability. Taken together, our findings suggest that heparanase inhibition protects against podocyte injury and may represent a potential therapeutic strategy for glomerular diseases.

## 1 Introduction

Chronic kidney disease (CKD) is a progressive renal insufficiency that can be indicated by high serum creatinine levels or the urinary loss of proteins (Leeuwis et al. 2010; Mundel and Shankland 2002). The filtration capacity of the glomerulus is defined by the functional properties of the glomerular filtration barrier (GFB). The GFB consists of three major layers: a unique capillary fenestrated endothelium, an intervening glomerular basement membrane (GBM), and the podocyte layer (Lal et al. 2015; Scott and Quaggin 2015). Podocytes are highly specialized glomerular epithelial cells with a unique structure, including a cell body, major processes, and foot processes (FPs). These FPs form a specialized interdigitating intercellular junction, known as the slit diaphragm, which covers the surface of the glomerular capillaries to prevent protein leakage during filtration (Brinkkoetter et al. 2013; Kriz and Lemley 2017). Nephrin, a transmembrane protein of the immunoglobulin family, is specifically located at the slit diaphram of glomerular podocytes (Ruotsalainen et al. 1999). It bridges the FPs of adjacent podocytes, serving as a structural backbone of the slit diaphragm, which is critical for maintaining both the structural and functional integrity of podocytes (Tryggvason 1999). Beyond its structural role, nephrin also functions as a signaling scaffold, facilitating the assembly of a protein complex that includes the scaffolding protein zonula occludens‐1 (ZO‐1), podocin, and CD2‐associated protein, which collectively link to the actin cytoskeleton and other signaling proteins beneath the cell membrane (Benzing 2004; Zhu et al. 2008).

The core structure of podocyte FPs is a well‐regulated actin cytoskeletal network, appearing either as dense actin bundles along the FP length to generate contractile forces or as short, branched structures at the edge to anchor the slit diaphragm (Mathieson 2012; Schell and Huber 2017; Welsh and Saleem 2012). Proper actin regulation in FPs is essential for slit diaphragm integrity and podocyte filtration function. Podocyte injury leads to proteinuria, the primary clinical manifestation of glomerulonephritis (Kopp et al. 2020; Nagata 2016). The hallmark of podocyte injury in biopsy is FP effacement, characterized by the loss of interdigitating FP structure accompanied by the deposition of actin filaments (Lennon et al. 2014; Mathieson 2012). Early FP effacement is reversible, but long‐term cumulative damage can result in podocyte detachment and loss. Since podocytes are post‐mitotic cells, their loss leads to irreversible glomerular injury (Kriz 2020; Nagata 2016). Maintaining podocyte adhesion and architecture is crucial for proper glomerular filtration function.

Podocytes adhere to the GBM through integrins, which initiate the formation of focal adhesions (FAs), multi‐protein complexes that physically and functionally connect the extracellular matrix (ECM) to the actin cytoskeleton (Sachs and Sonnenberg 2013). Upon ECM engagement, integrins recruit key adaptor proteins such as talin and paxillin to form nascent adhesions. These adhesions either disassemble or mature into stable FAs in an actomyosin contractility–dependent manner (Lennon et al. 2014; Schell and Huber 2017). The GBM is a dense network of ECM with a supramolecular assembly composed of type IV collagen, laminin, glycoproteins (such as entactin/nidogen), and various heparan sulfate (HS) proteoglycans (HSPG). This specialized niche provides a scaffold for endothelial cells and podocytes, facilitating the passage of water and solutes along a transcapillary pressure gradient (Naylor et al. 2021). In GBM, agrin is the primary proteoglycan that carries HS (Raats et al. 2000). It links laminin in the basement membrane to podocyte and endothelial surface adherent receptors. HS chains are important constituents and organizers of ECM, playing a key role in maintaining the GFB. Additionally, HS chains can selectively bind various growth factors and chemokines, contributing to numerous cellular processes (Bishop et al. 2007; Esko and Selleck 2002; Sarrazin et al. 2011; Taylor and Gallo 2006). Therefore, the regulation of HS turnover in the glomerulus is essential for maintaining cellular and tissue homeostasis (Rabelink et al. 2017; van den Hoven et al. 2007; van der Vlag and Buijsers 2020).

Heparanase is the only mammalian endoglycosidase known to degrade HS (Rabelink et al. 2017). It has been reported that heparanase is upregulated and activated in the glomeruli of rats with proteinuria caused by puromycin aminonucleoside (PAN)‐induced nephrosis (Levidiotis et al. 2001). A similar finding was observed in experimental models of Heymann nephritis, where the administration of a neutralizing antibody against heparanase or the heparanase inhibitor PI‐88 prevented the progression of glomerulonephritis (Levidiotis, Freeman, Punler, et al. 2004; Levidiotis, Freeman, Tikellis, et al. 2004). In addition, elevated levels of heparanase have been identified in various human proteinuric glomerular diseases, including diabetic nephropathy and different forms of glomerulonephritis (Gil et al. 2012; van den Hoven et al. 2006). Patients with these conditions exhibit increased urinary excretion of heparanase (Rops et al. 2007; Shafat et al. 2011). These findings underscore the critical role of heparanase in the progression of kidney disease with proteinuria and highlight its potential as a therapeutic target (Rabelink et al. 2017; van den Hoven et al. 2007; van der Vlag and Buijsers 2020).

The intricate regulation of the podocyte cytoskeleton, crucial for cell–cell and cell–matrix interactions, is fundamental to maintaining an intact GFB. However, whether podocytes exhibit heparanase activity during damage, contributing to their structural and functional defects, remains unclear. In this study, we utilized a conditionally immortalized mouse podocyte cell line to explore the role of heparanase in PAN‐induced podocyte injury, focusing on cytoskeletal structure and filtration function. Our findings indicate that heparanase inhibition effectively attenuates PAN‐induced podocyte injury.

## 2 Methods

### 2.1 Cell Line and Reagents

The conditional immortalized mouse podocyte line was kindly provided by Dr. Wen‐Chih Chiang (Division of Nephrology, Department of Internal Medicine, National Taiwan University Hospital, Taipei, Taiwan.) and originally from Dr. Peter Mundel (Drug Discovery, and Biology, Goldfinch Bio Inc., Cambridge, Massachusetts 02142, US) (Mundel, Reiser, et al. 1997). Heparanase inhibitor PI‐88 was provided by Medigen Biotechnology Corporation. HS sodium salt form bovine kidney (H7640), FITC‐BSA (A9771), and Hoechst (H33342) from Sigma‐Aldrich; type‐I collagen (#354236), type‐IV collagen (#354233), laminin/entactin complex (#354259) from Corning; anti‐synaptopodin antibody (NBP2‐39100) from Novus; anti‐nephrin (ab216341) and anti‐podocin (ab50339) antibodies from Abcam; anti‐GAPDH antibody (tcba13660) from Taiclone; anti‐paxillin (GTX125891) antibody from GeneTex; anti‐ZO‐1 (ZO1‐1A12), CF468A‐conjugated goat anti‐mouse and CF568A‐conjugated goat anti‐rabbit antibodies and CF633‐phalloidin (A22284) from Invitrogen; PAN (#15509) from Cayman; recombinant mouse IFNγ from BioLegend.

### 2.2 Cell Culture and Treatments

Podocytes expressing a temperature‐sensitive variant of the SV40 large T antigen under the control of the IFNγ‐inducible H‐2k^b^ promoter (Mundel, Reiser, et al. 1997) were cultured in RPMI 1640 medium supplemented with 10% heat‐inactivated fetal bovine serum (HI‐FBS), antibiotics (10 U/mL penicillin, 0.1 mg/mL streptomycin, and 0.25 µg/mL amphotericin B), and 10 U/mL IFNγ in a humidified atmosphere of 5% CO_2_/95% air at 33°C (Schiwek et al. 2004). To induce differentiation, podocytes were cultured in medium containing 2% HI‐FBS without IFNγ at 37°C on type‐I collagen‐coated dishes (10 µg/mL) for 10 to 12 days. For cell treatments, differentiated podocytes were re‐plated onto glass coverslips or Transwell inserts coated with an ECM mixture (20 µg/mL type‐IV collagen, 10 µg/mL type‐I collagen, 20 µg/mL laminin/entactin, and 1 µg/mL HS) and incubated in differentiation medium for 24 h. Podocytes were then treated with or without PAN (20, 40, or 100 µg/mL) and PI‐88 (25, 50, or 100 µg/mL) for 24 h in serum‐free medium.

### 2.3 Western Blot Analysis

Podocytes were lysed in lysis buffer containing 50 mM Tris‐HCl (pH 7.4), 150 mM NaCl, 1% NP‐40, 0.5% DOC, 0.1% SDS, 1 mM PMSF, and supplemented with a proteinase inhibitor cocktail (Sigma). Protein concentration was determined using the Bradford method. The lysates were heated to 100°C for 5 min in SDS gel‐loading buffer (50 mM Tris‐HCl, pH 6.8; 100 mM dithiothreitol; 2% SDS; 0.1% bromophenol blue; and 10% glycerol). Protein samples were separated by SDS polyacrylamide gel electrophoresis and transferred to a polyvinylidene difluoride (PVDF) membrane (Millipore). Membranes were then blocked with 5% nonfat dry milk in Tris‐buffered saline (TBS) with 0.1% Tween 20 for 1 h at room temperature. Blots were incubated overnight at 4°C with diluted primary antibodies: anti‐synaptopodin (1:500), anti‐nephrin (1:1000), anti‐podocin (1:1000), or anti‐GAPDH (1:50,000) in blocking buffer. The blots were then washed and incubated with horseradish peroxidase (HRP)‐conjugated goat anti‐rabbit IgG or anti‐mouse IgG antibodies to detect the primary antibodies. Enhanced chemiluminescence detection for the HRP reaction was performed according to the manufacturer's instructions.

### 2.4 Immunofluorescence Staining

Podocytes were fixed in 4% paraformaldehyde solution in phosphate‐buffered saline (PBS) for 30 min, washed, followed by permeabilization with 0.3% Triton X‐100 in TBS for 5 min. Samples were blocked with 5.5% normal goat serum for 30 min, and then probed with anti‐ZO‐1 (1:150) and anti‐paxillin (1:500) antibodies at 4°C overnight. Samples were then incubated with CF468A‐conjugated (1:100), CF568A‐conjugated (1:200) secondary antibodies, CF633‐phalloidin (1:600), and Hoechst (1 μg/mL) for 1 h, washed, mounted, and examined on a fluorescence microscope (Nikon Ti‐E), equipped with a 60x oil‐immersion objective lens. Image acquisition was performed using a cooled CCD digital camera (Hamamatsu ORCA‐ER), and image processing was carried out using ImageJ and Photoshop (Adobe) software.

### 2.5 Image Analysis

For FA size analysis, images of immunofluorescence staining using the anti‐paxillin antibody were segmented, and the FA area was quantified using ImageJ software. The number of small FAs (area < 0.5 μm²) and relatively mature FAs (area > 0.5 μm²) per individual cell was calculated and presented as scatter dot plots. To assess intercellular junction integrity, ZO‐1 staining patterns were categorized into two groups: normal and abnormal. Normal junctions were defined as (a) interdigitated lines or (b) discontinuous linear staining, while abnormal junctions were defined as (c) dot‐like staining accompanied by filamentous actin (F‐actin) accumulation or (d) complete loss of intercellular junctions. The percentage of cells displaying abnormal junctions (categories c and d) was calculated.

### 2.6 BSA Permeability Assay

The permeability assay was performed after 24 h of treatment by assessing BSA filtration across the podocyte monolayer (Delézay et al. 2017). Briefly, differentiated podocytes were seeded at a density of 5 × 10⁴ cells/cm² on ECM‐coated permeable Transwell inserts (Corning 3472, 24‐well, 3 μm pore size), and treated with or without PAN (40 μg/mL) and PI‐88 (0–100 μg/mL) in serum‐free medium for 24 h. After treatment, cells were washed with PBS and incubated in fresh serum‐free medium. FITC‐BSA (0.2 mg/mL) and endotoxin‐free BSA (10 mg/mL) were added to the bottom compartment (1 mL). To evaluate podocyte permeability in the basal‐to‐apical direction, 100 μL of medium was collected from the apical compartment after 2 h. The concentration of FITC‐BSA was measured using a fluorometer (Ex: 485 nm; Em: 538 nm) and calculated based on a standard curve.

### 2.7 RNA Extraction and RT‐qPCR

RNA extraction was performed using TRIzol (Invitrogen) and Direct‐zol RNA Miniprep Plus (Zymo Research) according to the manufacturer's protocol. RNA aliquots were reverse‐transcribed into cDNA using the HiScript I First Strand cDNA Synthesis Kit (Bionovus). mRNA levels were quantified by real‐time PCR using RealQ Plus 2X Master Mix Green (Ampliqon) according to the Two‐Step PCR program instructions. *SYNP* (NM_001109975.1) forward primer: 5′‐CTTTGGGGAAGAGGCCGATTG‐3′, reverse primer: 5′‐GTTTTCGGTGAAGCTTGTGC‐3′. *ATCB* (NM_007393.5) forward primer: 5′‐CATTGCTGACAGGATGCAGAAGG‐3′, reverse primer: 5′‐TGCTGGAAGGTGGACAGTGAGG‐3′. Data were analyzed using the relative quantification method ($2^{∆∆C_{t}}$), with *ATCB* expression serving as the reference to normalize cDNA inputs.

### 2.8 Statistical Analysis

Numerical data are presented as mean ± standard deviation (SD) and illustrated using scatter dot plots or bar graphs. The normality of data distribution within each group was assessed using the Shapiro–Wilk test. For comparison of *SYNPO* gene expression, a paired two‐tailed Student's *t*‐test was performed. For comparisons between two independent groups (e.g., image analysis or BSA filtration data), statistical significance was assessed using an unpaired two‐tailed *t*‐test with Welch's correction when the sample size was relatively large (e.g., ~30), and the Mann–Whitney *U*‐test (unpaired, non‐parametric) when the sample size was small. For comparisons among three or more groups, statistical significance was determined using one‐way ANOVA or Kruskal–Wallis test, depending on sample size and data normality.

## 3 Results

### 3.1 Cytoskeletal Architecture in Cultured Podocytes

**Figure 1:**
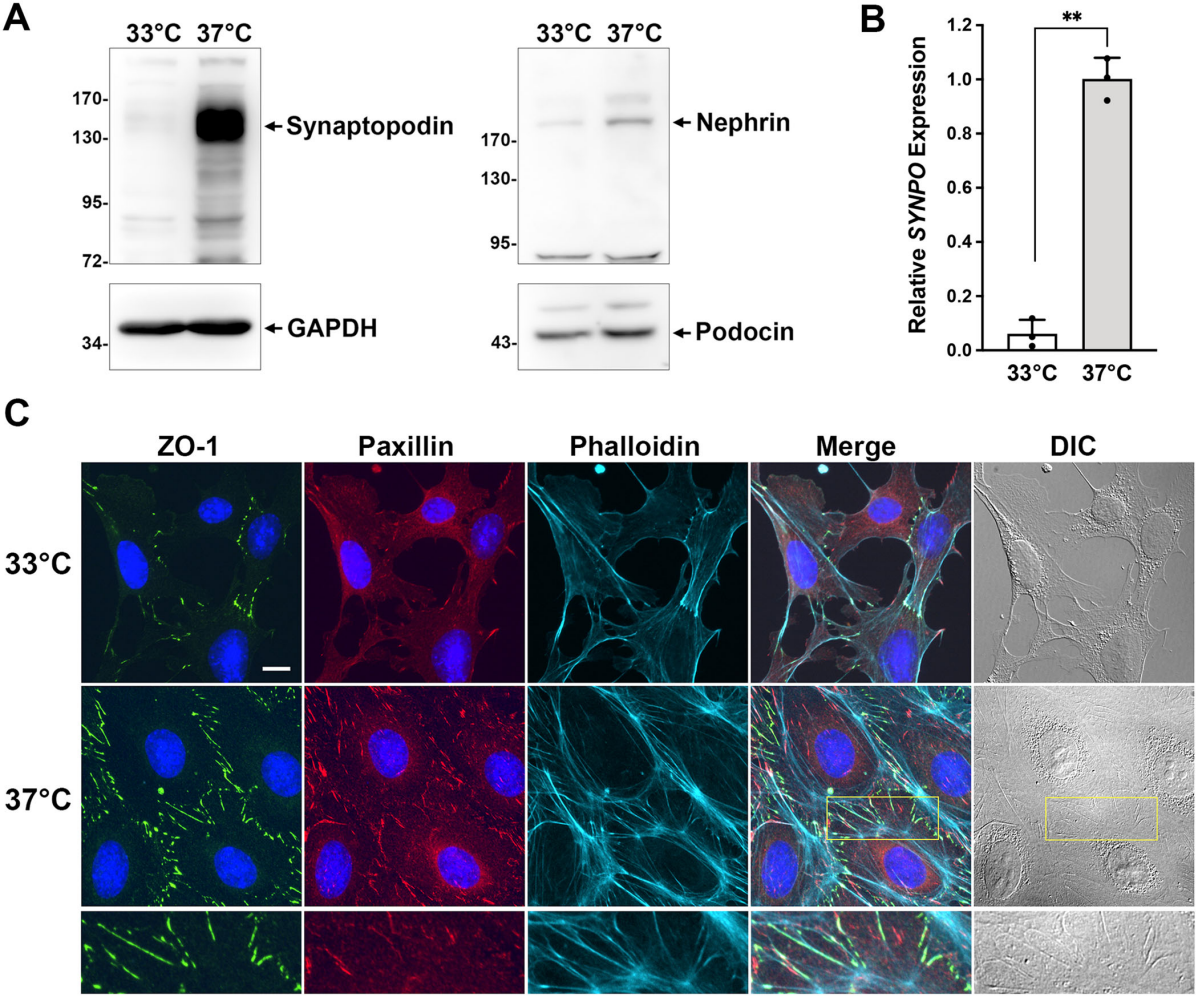
Temperature‐switch induction of mouse podocytes. Conditional immortalized mouse podocytes were cultured at 33°C in the presence of IFNγ (proliferative condition) or at 37°C in the absence of IFNγ (differentiated condition) for 13 days on the ECM‐coated substrates. (A) Cells were harvested for Western blot analysis using antibodies against synaptopodin, nephrin, and podocin, with GAPDH as an internal control. Original unprocessed blot images are provided in Figure S3. (B) Differential expression of the *SYNPO* gene was evaluated by RT‐qPCR. Data are presented as relative quantification, normalized to *ACTB* and to the 37°C differentiated podocytes (mean ± SD from three independent experiments). Raw *C*_t_ values are provided in Table S1. **p < 0.01 (paired, two‐tailed Student's *t*‐test). (C) Immunofluorescence staining of cells re‐plated on glass coverslips using anti‐paxillin (focal adhesions), anti‐ZO‐1 (intercellular junctions), phalloidin (F‐actin), and Hoechst (DNA). Scale bar, 10 μm.

To verify whether the inhibition of heparanase can directly exert a protective effect on podocytes, a conditional immortalized mouse podocyte cell line was utilized as a model (Schiwek et al. 2004). Cells were induced to differentiate by switching the temperature from 33°C (proliferative condition) to a nonpermissive 37°C (differentiated condition) in the absence of INFγ on type‐I collagen‐coated dishes for approximately 2 weeks. Podocyte differentiation was validated through the expression of mature podocyte marker synaptopodin, encoded by the *SYNPO* gene (Mundel, Heid, et al. 1997). Western blot analysis revealed a dramatic increase in the synaptopodin protein level in cells induced at 37°C (Figure 1A). The higher molecular weight of synaptopodin, approximately 130 kDa, observed in our data is consistent with other reports, suggesting possible posttranslational modification (Asanuma et al. 2005; Ning et al. 2020). Additionally, the expression of podocyte‐specific proteins such as nephrin and podocin was also detected (Figure 1A). RT‐qPCR analysis further confirmed the increased expression of *SYNPO* gene transcripts in cells induced at 37°C (Figure 1B and Table S1).

To observe and characterize the cytoskeletal architecture of cultured podocytes, cells were seeded onto glass coverslips coated with an ECM mixture containing type‐I collagen, type‐IV collagen, laminin/entactin, and HS, reflecting the composition of the GBM as described in the Materials and Methods. Immunofluorescent staining revealed that the formation of FAs and intercellular junctions, indicated by anti‐paxillin and anti‐ZO‐1 antibodies, respectively, was significantly enhanced in the 37°C differentiated cells. Notably, the intercellular junctions in the 37°C differentiated cells exhibited an interdigitating pattern, a key characteristic specific to podocytes. An increase in F‐actin was also observed, including both cortical actin and stress fibers (Figure 1C). These findings suggest that actomyosin contractility plays a crucial role in maintaining the cellular architecture of cultured podocytes, further supporting the successful differentiation of the cells at 37°C.

### 3.2 Assessing Cytoskeletal Changes in PAN‐Induced Podocyte Injury

**Figure 2:**
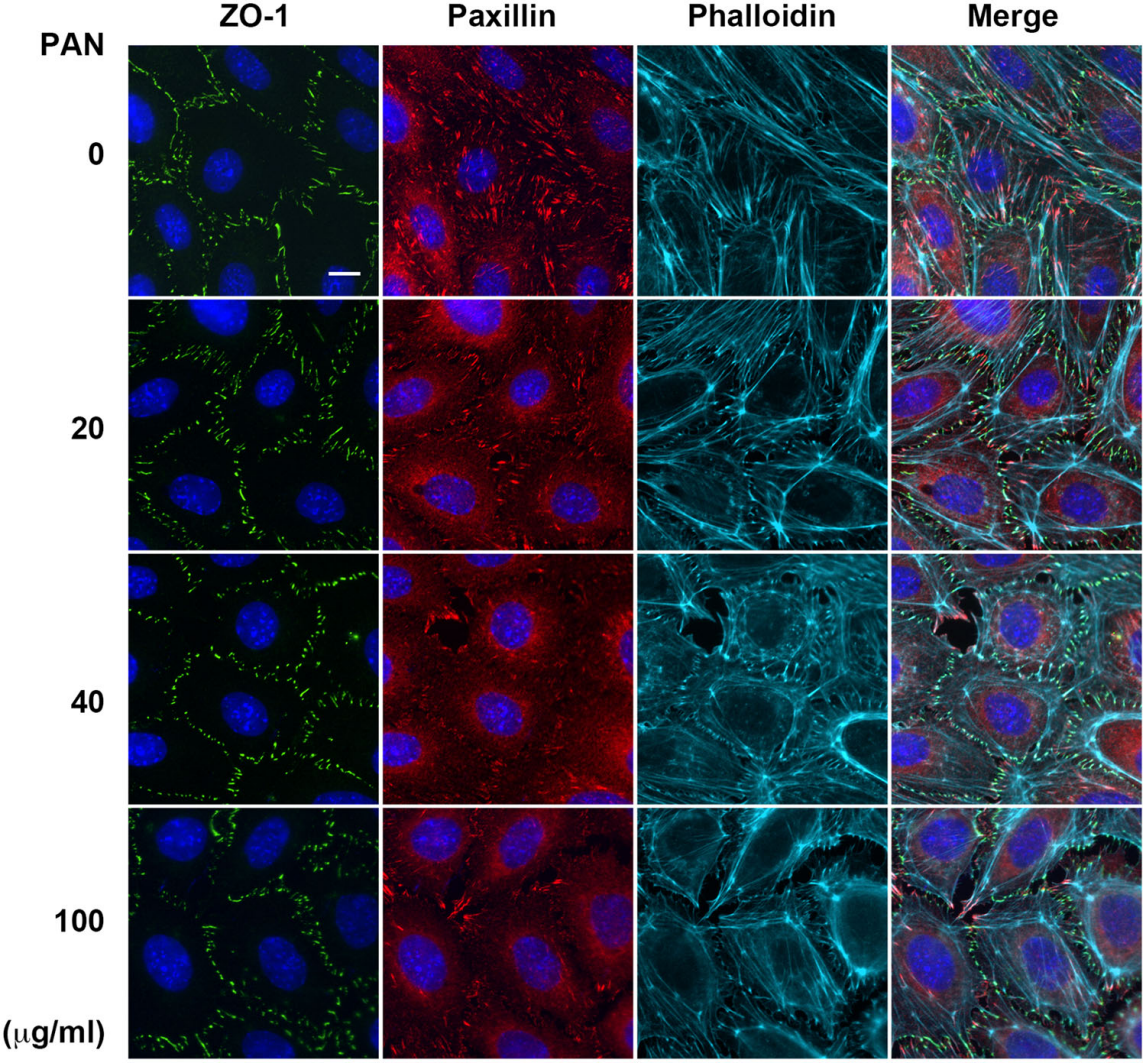
Alteration of cytoskeletal architecture in PAN‐induced podocyte injury. Differentiated podocytes were seeded on ECM‐coated glass coverslips and treated with ㄋㄧvarious concentrations of PAN for 24 h in serum‐free medium. Cells were fixed for immunofluorescence staining using anti‐paxillin (focal adhesions), anti‐ZO‐1 (intercellular junctions), phalloidin (F‐actin), and Hoechst (DNA). Scale bar, 10 μm.

PAN, a well‐known podocytotoxin, is widely used to study podocyte injury in vivo and in vitro in models of minimal change disease (MCD) (Marshall et al. 2006; Srivastava et al. 2013). Differentiated cultured podocytes were treated with various concentrations (0–100 μg/mL) of PAN for 24 h to induce podocyte injury (Figure 2). A significant reduction in FAs and stress fibers was observed in podocytes treated with 20 μg/mL PAN. This treatment also caused an enrichment of F‐actin intensity on actin filaments at the sites of cellular junctions, as observed by phalloidin and anti‐ZO‐1 staining. At a dose of 40 μg/mL PAN, the interdigitating intercellular junctions were almost effaced, transforming into dot‐like structures with strong F‐actin accumulation. Higher doses of PAN led to more severe disruption, causing a leaky, discontinuous cell monolayer. Cell density was quantified to assess podocyte viability, and no significant changes were observed across the different PAN concentrations tested for 24 h (Figure S1A). Thus, our data suggest that PAN‐induced changes in actin cytoskeleton and podocyte morphology were clearly distinguishable by microscopy imaging before any cell death occurred, with the extent of these changes varying by dose. Subsequent experiments were conducted using PAN at 40 μg/mL to determine the protective effects of heparanase inhibitor on podocyte injury.

### 3.3 The Protective Effect of PI‐88 on PAN‐Induced Podocyte Injury

**Figure 3:**
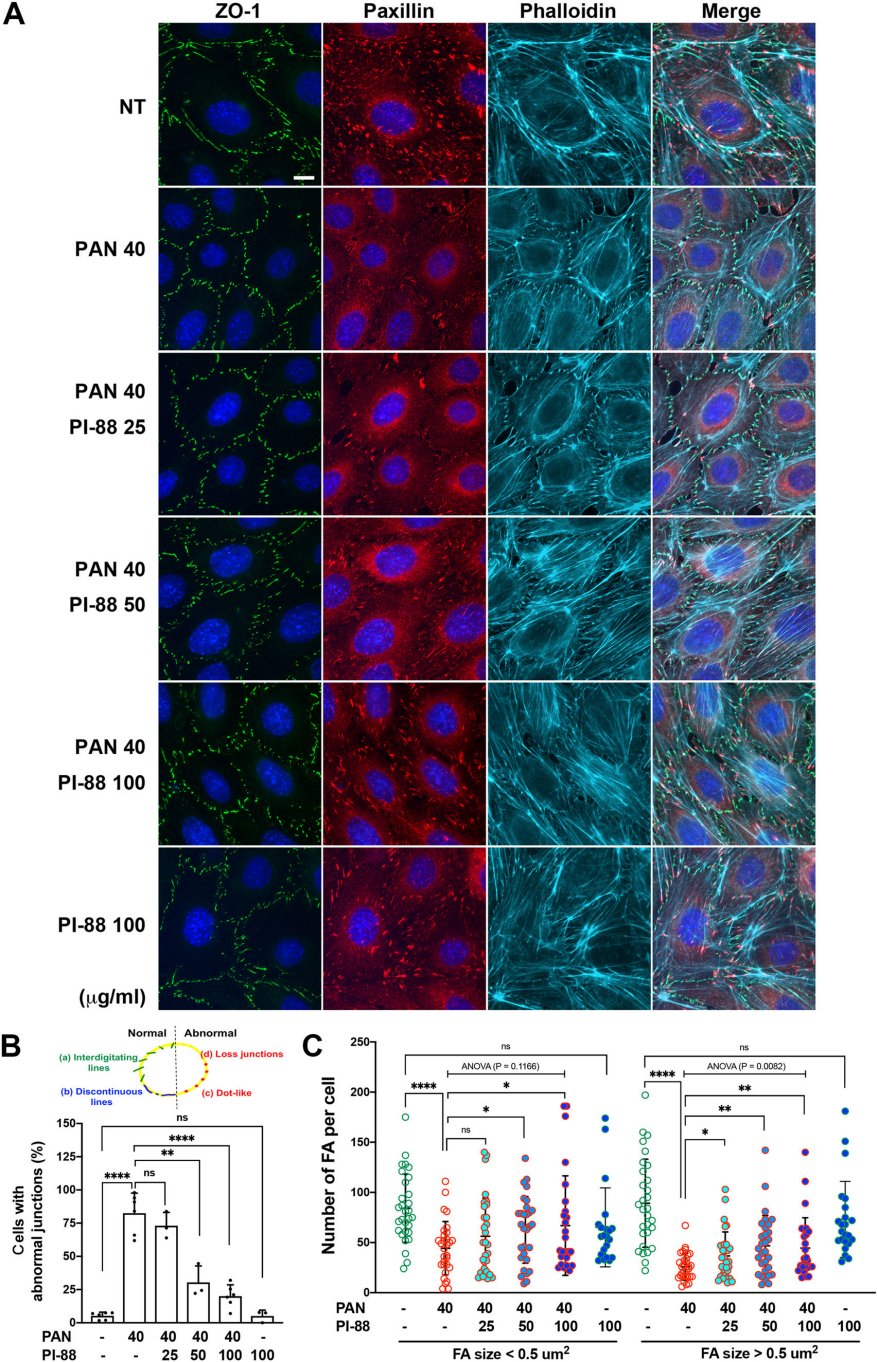
Inhibition of heparanase mitigates PAN‐induced podocyte architecture defects. Differentiated podocytes were cultured on ECM‐coated glass coverslips and treated with PAN (40 μg/mL) and PI‐88 (0–100 μg/mL) in serum‐free medium for 24 h. (A) Cells were fixed for immunofluorescence staining with anti‐paxillin (focal adhesions), anti‐ZO‐1 (intercellular junctions), phalloidin (F‐actin), and Hoechst (DNA). Scale bar, 10 μm. (B) Based on ZO‐1 staining phenotypes, intercellular junctions were categorized as either normal: (a) interdigitated lines or (b) discontinuous linear patterns; or abnormal: (c) dot‐like structures or (d) complete loss of junctions. The percentage of cells exhibiting abnormal junctions (c + d) was calculated. Data are expressed as mean ± SD from more than three independent experiments. Statistical comparisons between two groups were performed using the Mann–Whitney *U*‐test. (C) Scatter dot plots show the numbers of small FAs (area < 0.5 μm^2^) and relatively mature FAs (area > 0.5 μm^2^) per cell. Data are expressed as mean ± SD of approximately 30 representative cells from three independent experiments. Statistical comparisons between two groups (indicated by zigzag lines) were performed using an unpaired Welch's *t*‐test; for multiple groups comparisons (indicated by capped lines), ordinary one‐way ANOVA was used. *p < 0.05; **p < 0.01; ****p < 0.0001; ns, not statistically significant.

To examine whether the heparanase inhibitor PI‐88 has the protective potential against PAN‐induced podocyte injury, differentiated cultured podocytes seeded on the ECM‐coated glass coverslips were co‐treated with 40 μg/mL of PAN and various concentrations of PI‐88 for 24 h. As shown in Figure 3, PI‐88 co‐treatment demonstrated a protective effect on the cytoskeletal architecture of podocytes treated with PAN (Figure 3A), with no significant effect on cell density (Figure S1B). To quantify podocyte damage, we classified the status of intercellular junctions into two categories based on the anti‐ZO‐1 staining images. Healthy podocytes typically exhibit normal junctions as interdigitating or discontinuous lines, whereas damaged podocytes display abnormal junctions, such as dot‐like structures or complete loss of junctions. The percentage of cells with abnormal cellular junctions was calculated, showing that co‐treatment with PI‐88 dose‐dependently attenuated PAN‐induced defects in cellular junctions. Significant protection was observed at PI‐88 doses above 50 µg/mL (Figure 3B). We also quantified the numbers of small FAs (area < 0.5 μm²) and relatively more mature FAs (area > 0.5 μm²) in these podocytes. Our data showed that co‐treatment with PI‐88 at concentrations above 25 μg/mL effectively restored the number of mature FAs in PAN‐injured podocytes. In contrast, small FAs were less significantly affected and showed a statistically significant increase only at concentrations exceeding 50 μg/mL (Figure 3C). These results indicate that the effect of PI‐88 on restoring mature FAs is more pronounced than its effect on small FAs. Additionally, treatment with PI‐88 alone, even at higher concentrations (up to 400 μg/mL), did not exert any deleterious effects on podocyte morphology (Figure S2). Together, these findings suggest that co‐treatment with the heparanase inhibitor PI‐88 attenuates PAN‐induced cytoskeletal abnormalities, thereby supporting the maintenance of podocyte attachment and structural integrity.

**Figure 4:**
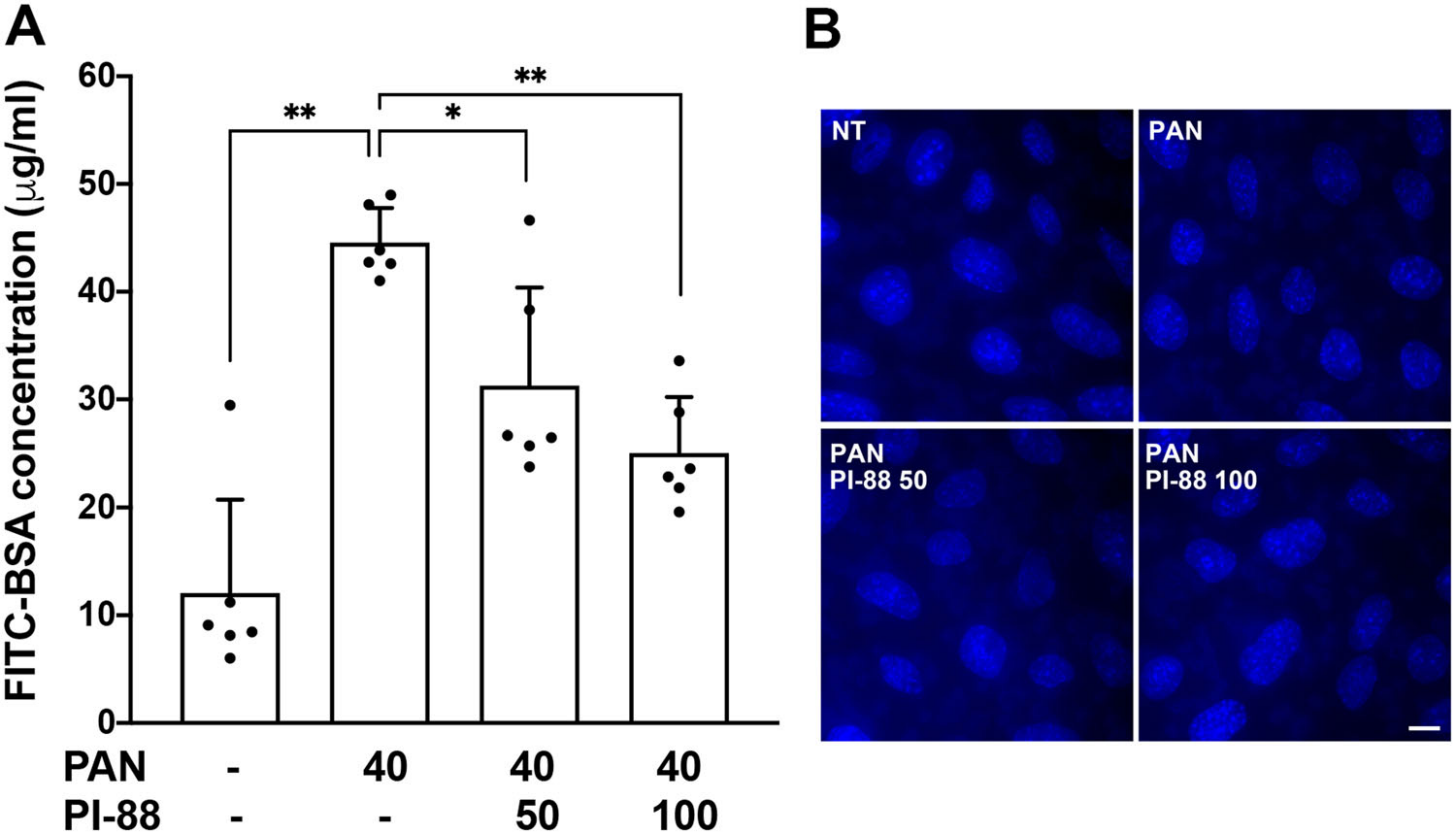
PI‐88 treatment rescues podocyte filtration function during PAN‐induced injury. Differentiated podocytes were cultured on the ECM‐coated Transwell inserts (3 μm pore size) and treated with or without PAN and PI‐88 as indicated, in serum‐free medium for 24 h. FITC‐BSA filtration assays were then performed. (A) The concentration of FITC‐BSA in the apical compartment was measured after 2 h of incubation. Data are presented as mean ± SD (*n* = 6). Statistical comparisons between two groups were performed using the Mann–Whitney *U*‐test. *p < 0.05; **p < 0.01. (B) Cell density on the Transwell membrane was visualized by Hoechst staining. Scale bar, 10 μm.

To confirm the protective effects of PI‐88 against PAN‐induced podocyte injury, podocyte function was assessed using the FITC‐BSA filtration assay. Differentiated podocyte monolayers were cultured on ECM mixture‐coated Transwell membranes with a pore size of 3.0 μm. Podocytes were then treated in serum‐free medium as indicated and subsequently subjected to the FITC‐BSA filtration assay. The permeability of FITC‐BSA diffusion from the basal to the apical compartment was determined as described in the Methods section. PAN treatment significantly increased FITC‐BSA permeability, indicating compromised barrier function of the podocyte monolayer. In contrast, co‐treatment with PI‐88 reduced FITC‐BSA leakage, supporting a protective role of heparanase inhibition in PAN‐induced podocyte injury (Figure 4). These functional findings are consistent with the structural improvements observed in cytoskeletal organization and intercellular junction integrity upon PI‐88 treatment. In conclusion, PI‐88 offered protective potential against PAN‐induced podocyte damage, preserving both cytoskeletal architecture and filtration function.

## 4 Discussion

Podocyte function relies on its unique cytoskeletal architecture to maintain the complex morphology essential for its role in the kidney filtration barrier (Mathieson 2012; Schell and Huber 2017; Welsh and Saleem 2012). Podocyte damage results in proteinuria, a common early clinical manifestation in various glomerulopathies and a key factor in the progression of CKD (Meliambro et al. 2024; Nagata 2016). In this study, we used differentiated cultured podocytes to model PAN‐induced injury. Podocyte damage was assessed through immunofluorescence staining to observe cytoskeletal changes, and a BSA permeability assay was used to evaluate filtration function. Our results demonstrate that co‐treatment with the heparanase inhibitor PI‐88 significantly mitigated cytoskeletal damage and BSA leakage in PAN‐treated podocytes. These findings highlight the detrimental effects of excessive heparanase activity on podocytes and underscore its potential as a therapeutic target in podocyte injury.

HSPGs are located in the ECM, particularly in basement membranes, and on cell surfaces, where they play crucial roles in cell–cell and cell–matrix adhesion. Heparanase, the only known endoglucuronidase, cleaves specific internal glycosidic bonds within HS chains of HSPGs, releasing smaller HS fragments. The degradation of HSPGs by heparanase may also result in the release of ligands, including growth factors, chemokines, and cytokines stored in the GBM, thereby affecting various physiological and pathological processes (Rabelink et al. 2017; van den Hoven et al. 2007; van der Vlag and Buijsers 2020). It has been proposed that excessive glomerular heparanase activity leads to the loss of HS in the GBM, altering its charge‐selective permeability and podocyte–GBM interactions (van den Hoven et al. 2007). This hypothesis is supported by evidence from animal experimental models and clinical observations, which show increased heparanase levels associated with HS loss in various proteinuric diseases, including diabetic nephropathy, MCD, IgA nephropathy, lupus nephritis, and membranous nephropathy (Lever et al. 2014; Rops et al. 2004; Tamsma et al. 1994; van den Born et al. 1992, 1993). Notably, elevated heparanase activity has been detected in the urine of proteinuric patients (Katz et al. 2002; Shafat et al. 2011). Additionally, transgenic mice overexpressing heparanase developed mild proteinuria (van den Hoven et al. 2006). These studies underscore the role of HS degradation from the GBM by heparanase in the development of proteinuria in nephropathy.

However, conflicting findings presenting alternative perspectives challenge the notion that HS degradation in the GBM is directly responsible for proteinuria. Structural analysis of glomerular HS in kidney biopsies from type I diabetic patients with early albuminuria, as well as in rat renal tissue after 5 months of diabetes, revealed no significant changes in HS (van den Born et al. 2006). Similarly, in vivo degradation of GBM HS by heparinase did not induce albuminuria (van den Hoven et al. 2008; Wijnhoven et al. 2007). Furthermore, podocyte‐specific agrin knockout mice, which lack HS in the GBM, also failed to develop proteinuria, despite reduced GBM anionic charge (Harvey et al. 2007). Podocyte‐specific knockout of EXT‐1 in mice, which lack the enzyme required for HS polymerization, did not develop severe albuminuria, even though podocyte abnormalities and HS loss were observed (Chen et al. 2008). These findings suggest that the loss of HS alone is insufficient to cause proteinuria, and additional mechanisms beyond HS degradation may contribute to the development of albuminuria.

In this study, we demonstrated the involvement of heparanase in PAN‐induced podocyte injury by showing that the heparanase inhibitor PI‐88 protected against cytoskeletal defects and BSA leakage in cultured podocytes. Under our experimental conditions, podocytes were cultured on substrates coated with various commercial ECM molecular mixtures, as described in the Methods. These mixtures were specifically designed to exclude chemokines, cytokines, and other ligands. Furthermore, in our previous study (Tseng et al. 2022), podocytes displayed an intact cytoskeleton, including mature FAs and interdigitating intercellular junctions, when attached to a substrate coated solely with type IV collagen. Since type IV collagen alone is unlikely to possess HS linkages (Naylor et al. 2021), this suggests that the presence of HS in the ECM is not required for podocyte adhesion. Consequently, the protective effects of the heparanase inhibitor observed here are unlikely to result from inhibiting HS degradation or releasing factors from the ECM. In addition to the GBM, HSPGs are found on the surfaces of various cells, such as endothelial cells and leukocytes. Heparanase‐mediated cleavage of cell surface HS has been reported to play a critical role in regulating inflammation, particularly during the progression of glomerular diseases (Garsen et al. 2016; Gilat et al. 1995; Lever et al. 2014; Rops et al. 2004). However, the mechanisms through which heparanase influences podocyte injury remain unclear, especially in contexts involving complex multiorgan and cellular interactions. In this study, PI‐88 protected cultured podocytes from PAN‐induced injury in vitro. This suggests that heparanase activity is necessary for the processes leading to podocyte injury, and the podocytes themselves likely produce the heparanase substrate.

In conclusion, we utilized in vitro differentiated cultured podocytes and induced damage using PAN treatment, resulting in quantifiable cytoskeletal defects and increased BSA permeability. We demonstrated that co‐treatment with PI‐88, a heparanase inhibitor, prevented PAN‐induced injury. Our data clearly support the involvement of heparanase in podocyte injury and suggest the existence of an autocrine deleterious mechanism triggered by heparanase in podocytes. Further studies are required to clarify the detailed molecular mechanisms underlying these findings.

## Author Contributions

X.‐Y.H. and Y.‐H.L. conducted the experiments and performed the data analysis. H.‐H.L. was responsible for designing the research, analyzing the results, and writing the manuscript. During the preparation of this study, the author(s) used Grammarly and ChatGPT to check English text for spelling, grammar, and delivery errors. After using this tool/service, the author(s) reviewed and edited the content as needed and take(s) full responsibility for the content of the publication.

## Conflicts of Interest

The authors declare no conflicts of interest.

## Supporting information

HPSEi_JCP_Supplemental.

## Data Availability

The data that support the findings of this study are available from the corresponding author upon reasonable request.
