# Supplementary material for: Targeting Heparanase Attenuates Podocyte Injury Induced by Puromycin Aminonucleoside
Source: J Cell Physiol. 2025 Jun 10;240(6):e70053. doi: 10.1002/jcp.70053 (PMC12152518; doi:10.1002/jcp.70053)
Supplement: Supplementary file 1 — HPSEi_JCP_Supplemental. [file JCP-240-0-s001.docx]

**Supplemental Table**

**Table S1.** Ct values of *SYNPO* and *ACTB* genes in mouse podocytes under proliferative and differentiated conditions.

| **Gene** | **Condition** | **Mean Ct ± SD** |
| --- | --- | --- |
| ***SYNPO*** | 33°C (proliferative) | 31.92 ± 1.12 |
|  | 37°C (differentiated) | 27.53 ± 0.10 |
| ***ACTB*** | 33°C (proliferative) | 14.97 ± 0.13 |
|  | 37°C (differentiated) | 15.06 ± 0.10 |

Ct values obtained from RT-qPCR analysis of *SYNPO* and *ACTB* gene expression in conditional immortalized mouse podocytes cultured under proliferative (33°C with IFNγ) or differentiated (37°C without IFNγ) conditions for 13 days. Data are presented as mean ± SD from three independent experiments.

**Supplementary Figures**


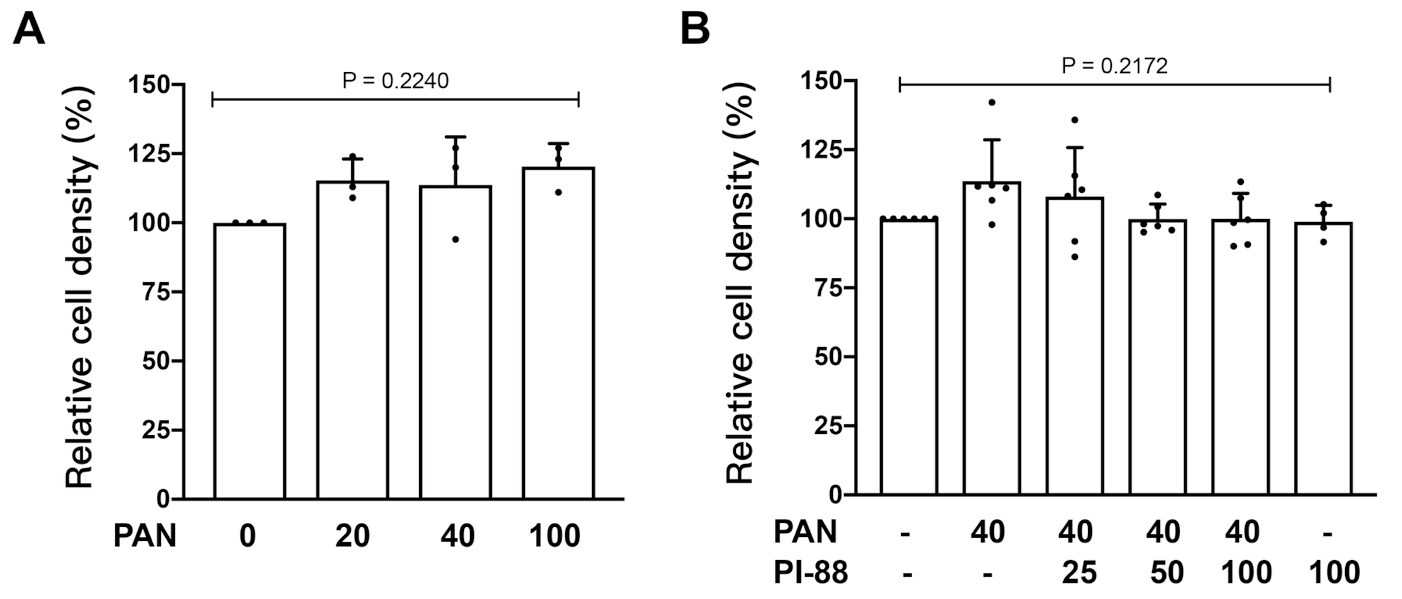


**Figure S1. Relative cell density of podocytes under various treatment conditions.**

Differentiated podocytes were seeded on ECM-coated glass coverslips and treated for 24 hours in serum-free medium with either (A) various concentrations of PAN, or (B) co-treatment with PAN and PI-88 as indicated. Cell density was assessed using Hoechst-stained images. Data are presented as relative quantification compared to the non-treated control (mean ± SD). No statistically significant differences were observed among the treatment groups (Kruskal–Wallis test).


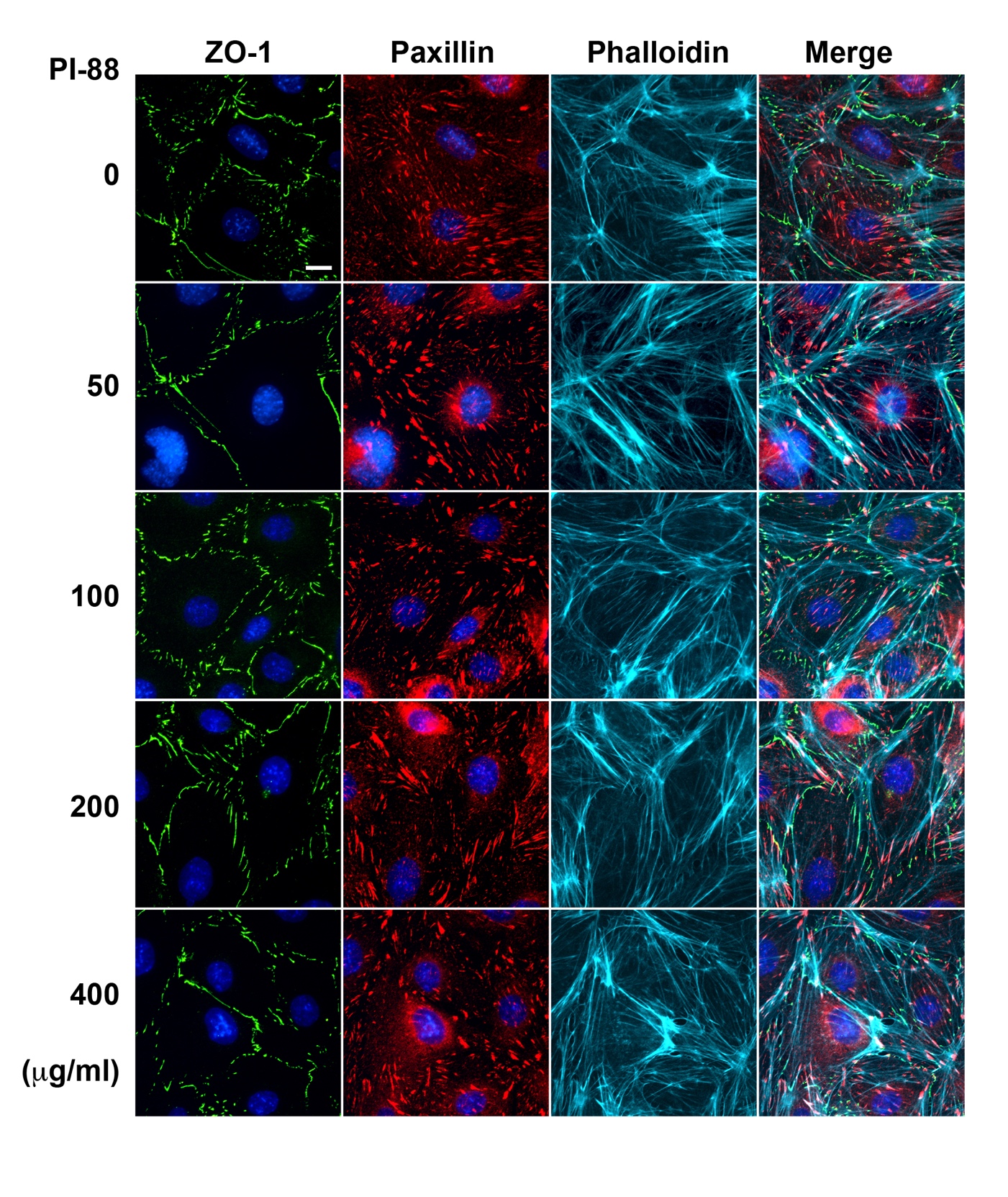


**Figure S2. PI-88 has no significant deleterious effect on the cytoskeleton of podocytes.**

Differentiated podocytes were cultured on ECM-coated coverslips and treated with PI-88 (0-400 μg/ml) in serum free medium for 24 hr. Cells were fixed for immunofluorescence staining using anti-paxillin (focal adhesions), anti-ZO-1 (intercellular junctions), phalloidin (F-actin), and Hoechst (nuclei). Bar, 10 μm.


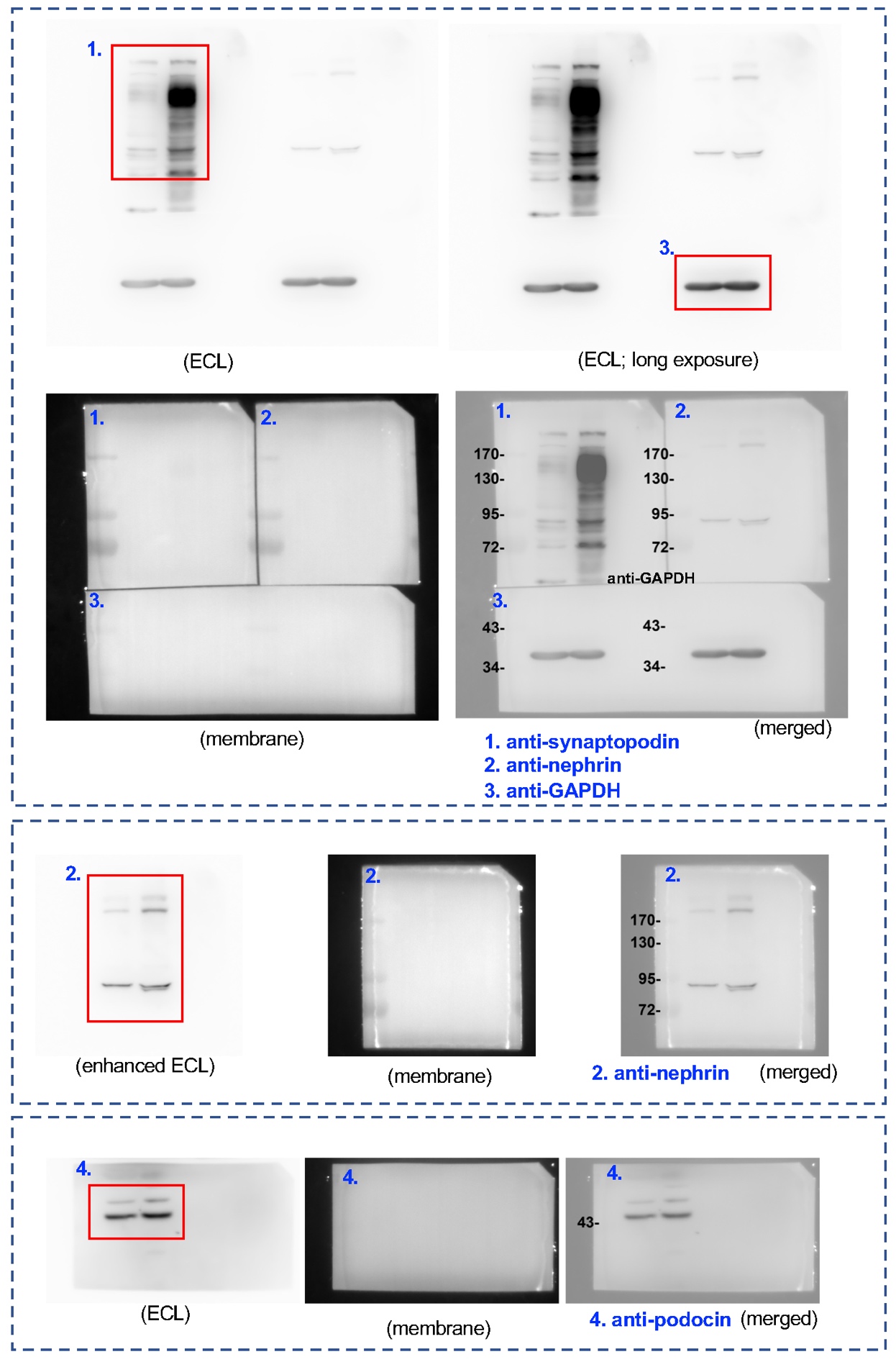


**Figure S3. Source data in Figure 1A.**

Unprocessed original images used in Figure 1A are provided, including the ECL images, corresponding membrane images, and their merged views.
